# Development and validation a nomogram for predicting the risk of severe COVID-19: A multi-center study in Sichuan, China

**DOI:** 10.1371/journal.pone.0233328

**Published:** 2020-05-18

**Authors:** Yiwu Zhou, Yanqi He, Huan Yang, He Yu, Ting Wang, Zhu Chen, Rong Yao, Zongan Liang

**Affiliations:** 1 Department of Emergency Medicine, Emergency Medical Laboratory, West China Hospital, Sichuan University, Chengdu, Sichuan, China; 2 Disaster Medical Center, Sichuan University, Chengdu, Sichuan, China; 3 Department of Respiratory and Critical Care Medicine, West China Hospital, Sichuan University, Chengdu, China; 4 Public Health Clinical Center of Chengdu, Chengdu, China; Osaka University Graduate School of Medicine, JAPAN

## Abstract

**Background:**

Since December 2019, coronavirus disease 2019 (COVID-19) emerged in Wuhan and spread across the globe. The objective of this study is to build and validate a practical nomogram for estimating the risk of severe COVID-19.

**Methods:**

A cohort of 366 patients with laboratory-confirmed COVID-19 was used to develop a prediction model using data collected from 47 locations in Sichuan province from January 2020 to February 2020. The primary outcome was the development of severe COVID-19 during hospitalization. The least absolute shrinkage and selection operator (LASSO) regression model was used to reduce data size and select relevant features. Multivariable logistic regression analysis was applied to build a prediction model incorporating the selected features. The performance of the nomogram regarding the C-index, calibration, discrimination, and clinical usefulness was assessed. Internal validation was assessed by bootstrapping.

**Results:**

The median age of the cohort was 43 years. Severe patients were older than mild patients by a median of 6 years. Fever, cough, and dyspnea were more common in severe patients. The individualized prediction nomogram included seven predictors: body temperature at admission, cough, dyspnea, hypertension, cardiovascular disease, chronic liver disease, and chronic kidney disease. The model had good discrimination with an area under the curve of 0.862, C-index of 0.863 (95% confidence interval, 0.801–0.925), and good calibration. A high C-index value of 0.839 was reached in the interval validation. Decision curve analysis showed that the prediction nomogram was clinically useful.

**Conclusion:**

We established an early warning model incorporating clinical characteristics that could be quickly obtained on admission. This model can be used to help predict severe COVID-19 and identify patients at risk of developing severe disease.

## Introduction

Since December 2019, several cases of coronavirus disease (COVID-19) emerged in Wuhan, Hubei Province, China[1–3]. SARS-Cov-2 is transmitted by respiratory droplets and aerosols, direct contact, and stool[8, 9]. COVID-19 is very contagious, and the general population is highly susceptible to infection. The number of affected countries and number of deaths have increased dramatically since the beginning of the outbreak[4]. COVID-19 infection reached more than 130 countries. According to the World Health Organization, as of April 17, 2020, more than 2,034,802 confirmed cases were reported worldwide, and more than 135,163 infected patients died[5]. Therefore, COVID-19 is a serious health problem worldwide.

Coronavirus can affect multiple organs, including the lungs[1]. The main clinical presentation of COVID-19 is pneumonia. Most patients have mild disease, with common respiratory symptoms and good prognosis[6]. The most common clinical symptoms are fever and cough. However, it has been reported that only 43.8% of patients present with fever on admission. Radiologic abnormalities were not observed on initial presentation in approximately 20% of cases[7]. According to the sixth edition of the Novel Coronavirus Pneumonia Diagnosis and Treatment Plan, severe cases should meet any of the following criteria: 1) shortness of breath (respiratory rate ≥30 breaths per min), 2) oxygen saturation ≤93% at rest, or 3) arterial partial pressure of oxygen/fraction of inspired oxygen ≤ 300 mm Hg[8]. A small percentage of patients present with severe disease before or during hospitalization, including severe pneumonia, adult respiratory distress syndrome, or multiple organ failure, which are associated with worse outcomes[9–11]. As of February 15, 2020, the mortality rate and percentage of severe cases in Hubei Province were 2.6% and 15.2%, respectively[11, 12]. Therefore, specific predictive methods are urgently required to predict the risk of severe COVID-19[9]. Of all existing models, nomogram allows for individualized and evidence-based risk estimation, facilitating management-based decision-making[13, 14]. To the best of our knowledge, no previous studies have evaluated early warning models for predicting the risk of severe COVID-19.

Previous studies have shown that there are significant regional differences in the mortality rate and percentage of severe cases[12, 15]. The aim of this study is to describe the clinical characteristics of confirmed cases of COVID-19 in Sichuan, China, and construct an early warning prediction nomogram model incorporating clinical characteristics to identify the risk of developing severe COVID-19.

## Patients and methods

### Study design and participants

This retrospective, multicenter study included consecutive patients with laboratory-confirmed cases of COVID-19 in Sichuan, China, reported by the National Health Commission between January 20, 2019, and February 8, 2020. The data cutoff for the study was February 22, 2020. COVID-19 was confirmed by high-throughput sequencing or real-time reverse-transcriptase-polymerase-chain-reaction (RT-PCR) of nasal and pharyngeal swab specimens from the upper respiratory tract[16]. All study patients were diagnosed with COVID-19 according to the WHO interim guidance[17]. The study was approved by the Research Ethics Committee of West China Hospital, and data were collected retrospectively after patients gave written informed consent.

### Demographical and risk variables

The following data were obtained from electronic medical records: demographics (age and gender), clinical signs on admission, clinical symptoms, clinical risk factors, and exposure to infection. Clinical symptoms were defined as the interval between the onset of clinical symptoms and the data of admission. Exposure to infection was defined as contact with sources of infection in the past 14 days, including Wuhan or other COVID-19 affected areas, febrile patients, or COVID-19 patients, and the incidence had clustering phenomenon. The risk of exposure to infection changed as the relevant definitions in the COVID-19 guidelines of the National Health Commission of the People’s Republic of China changed. National early warning score (NEWS)[18] was calculated on admission. If data were missing from the records or clarification was needed, data were obtained by direct communication with attending physicians and other health care providers. All data were analyzed by two physicians (He YQ and Zhou YW), and a third researcher was consulted in cases of disagreement. The clinical and demographic features in our cohort are summarized in Table 1.

**Table 1:** Baseline characteristics of patients with COVID-19.

	Disease severity (No. %)			
	Total (N = 366)	Mild (N = 323)	Severe (N = 43)	P-value
Age (years)				
Median (IQR)	43 (31.8–51.0)	42 (31–50)	48 (37–64)	0.001
18–64	330 (90.2)	295 (91.3)	35 (81.4)	0.054
≥65	36 (9.8)	28 (8.7)	8 (18.6)	
Gender				
Female	159 (43.4)	142 (44.0)	17(39.5)	0.582
Male	207 (56.6)	181 (56.0)	26 (60.5)	
Heart rate, beats per minute				
< 90	299 (81.7)	263 (81.4)	36 (83.7)	0.714
≥ 90	67 (18.3)	60 (18.6)	7 (16.3)	
Respiratory rate, breaths per minute				
<25	292 (79.8)	255 (78.9)	37 (86.0)	0.276
≥ 25	74 (20.2)	68 (21.1)	6 (14.0)	
Systolic blood pressure, mmHg				
<110	29 (7.9)	28 (8.7)	1 (2.3)	0.228
≥ 110	337 (92.1)	295 (91.3)	42 (97.7)	
Body temperature on admission, °C				
≤ 37.2	258 (70.5)	224 (69.3)	34 (79.1)	0.322
37.3–38.0	37 (10.1)	33 (10.2)	4 (9.3)	
>38.0	71 (19.4)	66 (20.5)	5 (11.6)	
NEWS score				
<4	276 (75.4)	246 (76.2)	30 (69.8)	0.360
4–7	90 (24.6)	77 (23.8)	13 (30.2)	
Oxygen saturation, SpO_2_ (%)				
≥ 96	302 (82.5)	269 (83.3)	33 (76.7)	0.289
<96	64 (17.5)	54 (16.7)	10 (23.3)	
**Symptoms**				
Fever				
Yes	157 (42.9)	132 (40.9)	25 (58.1)	0.032
No	209 (57.1)	191 (59.1)	18 (41.9)	
Cough				
Yes	115 (31.4)	85 (26.3)	30 (69.8)	0.000
No	251 (68.6)	238 (73.7)	13 (30.2)	
Dyspnea				
Yes	23 (6.3)	11 (3.4)	12 (27.9)	0.000
No	343 (93.7)	312 (96.6)	31 (72.1)	
Chest pain				
Yes	5 (1.4)	4 (1.2)	1 (2.3)	0.467
No	361 (98.6)	319 (98.8)	42 (97.7)	
Fatigue				
Yes	26 (7.1)	24 (7.4)	2 (4.7)	0.753
No	340 (92.9)	299 (92.6)	41 (95.3)	
Muscle and joint pain				
Yes	14 (3.8)	13 (4.0)	1 (2.3)	1.000
No	352 (96.2)	310 (96.0)	42 (97.7)	
Digestive symptoms				
Yes	27 (7.4)	27 (8.4)	0 (0)	0.057
No	339 (92.6)	296 (91.6)	43 (100)	
Nervous symptoms				
Yes	16 (4.4)	14 (4.3)	2 (4.7)	1.000
No	350 (95.6)	309 (95.7)	41 (95.3)	
**Comorbidities**				
Diabetes				
Yes	21 (5.7)	15 (4.6)	6 (14.0)	0.026
No	345 (94.3)	308 (95.4)	37 (86.0)	
Hypertension				
Yes	38 (10.4)	24 (7.4)	14 (32.6)	0.000
No	328 (89.6)	299 (92.6)	29 (67.4)	
Cardiovascular disease				
Yes	9 (2.5)	2 (0.6)	7 (16.3)	0.000
No	357 (97.5)	321 (99.4)	36 (83.7)	
COPD				
Yes	10 (2.7)	7 (2.2)	3 (7.0)	0.101
No	356 (97.3)	316 (97.8)	40 (93.0)	
Chronic liver disease				
Yes	8 (2.2)	4 (1.2)	4 (9.3)	0.008
No	358 (97.8)	319 (98.8)	39 (90.7)	
Cerebrovascular disease				
Yes	4 (1.1)	4 (1.2)	0 (0)	1.000
No	362 (98.9)	319 (98.8)	43 (100)	
Chronic kidney disease				
Yes	4 (1.1)	1 (0.3)	3 (7.0)	0.006
No	362 (98.9)	322 (99.7)	40 (93.0)	
Malignancy				
Yes	1 (0.3)	1 (0.3)	0 (0)	1.000
No	365 (99.7)	322 (99.7)	43 (100)	
**Exposure to sources of infection in the past 14 days**				
Recently visited Wuhan or other COVID-affected area				
Yes	61 (16.7)	45 (13.9)	16 (37.2)	0.000
No	305 (83.3)	278 (86.1)	27 (62.8)	
History of contact with febrile patients				
Yes	13 (3.6)	11 (3.4)	2 (4.7)	0.656
No	353 (96.4)	312 (96.6)	41 (95.3)	
History of contact with COVID-19 patients				
Yes	23 (6.3)	23 (7.1)	0 (0)	0.091
No	343 (93.7)	300 (92.9)	43 (100)	
Clustering phenomenon				
Yes	18 (4.9)	18 (5.6)	0 (0)	0.147
No	348 (95.1)	305 (94.4)	43 (100)	

IQR, interquartile range; COPD, chronic obstructive pulmonary disease; NEWS, national early warning score.

### Definition of outcomes

The primary outcome was severe COVID-19 during hospitalization according to the American Thoracic Society guidelines for community-acquired pneumonia[19]. Severe cases should meet one major criterion (septic shock with need for vasopressors or respiratory failure requiring mechanical ventilation) or at least three minor criteria (respiratory rate ≥30 breaths per min, arterial partial oxygen pressure/fraction of inspired oxygen ≤250 mmHg, multilobar infiltrates, confusion/disorientation, uremia (blood urea nitrogen level ≥20 mg/dL), leukopenia (white blood cell count <400 cells/μL), thrombocytopenia (platelet count <100,000/μL), hypothermia (core temperature <36°C), and hypotension requiring aggressive fluid resuscitation[19].

### Statistical analysis

Statistical analyses were performed using R software version 3.5.1 (R Foundation for Statistical Computing, Vienna, Austria) and SPSS version 25.0 (IBM Corporation, Armonk, NY). Continuous variables were expressed as median and interquartile range. Categorical variables were expressed as absolute values and percentages. The means of continuous variables were compared using independent group *t*-tests for normally distributed data and the Mann-Whitney test for non-normally distributed data. The χ^2^ or Fisher’s exact test was used to compare proportions.

The least absolute shrinkage and selection operator (LASSO) method, which is suitable for analyzing high-dimensional data, was used to select the most significant predictive features[20, 21]. Features with non-zero coefficients in the LASSO regression model were selected in the forward stepwise logistic regression model[22]. The features were considered as odds ratio (OR) with 95% confidence interval [23] and two-tailed p-values. Variables with p-values smaller than 0.1 in the univariate analysis and potentially significant in the multivariate analysis were included in the logistic regression analysis, and the forward selection procedure was used to develop a parsimonious model for predicting severe COVID-19 in our cohort.

Nomogram is a statistical model useful for risk assessment. A predictive nomogram was developed using the independent factors selected by LASSO to generate a combined indicator for estimating the severity of COVID-19, and provided a quantitative tool for physicians to assess the individual probability of disease severity. The created nomogram was used for internal validation, and the total score for each nodule was calculated. The nomogram was constructed using the total score as a factor. Adequate discrimination and calibration were performed to test and validate the prognostic accuracy of the nomogram model[24]. Discrimination was quantified using Harrell’s concordance index (C-index), in which an absolute value close to 1 indicated that the model had strong predictive ability. The nomogram was further validated by bootstrapping (1000 bootstrap replicates) to calculate the corrected C-index. Calibration plots were developed to assess the predictive accuracy and agreement between predicted and observed severity. Decision curve analyses (DCAs) were performed to assess the clinical usefulness of the nomogram. The net benefit was calculated by subtracting the proportion of patients with false-positive results from the proportion of patients with true-positive results and by weighing the relative risk of an intervention compared with the adverse effects of an unnecessary intervention. The precision of the predictions was evaluated using the area under the receiver-operating characteristic curve (AUC). Two-sided p-values of less than 0.05 were considered to indicate a statistically significant difference.

## Results

### Clinical characteristics of patients

A total of 366 patients with COVID-19 who had been hospitalized in 47 regions of Sichuan were enrolled until January 20, 2020. Most patients were admitted to a public health clinical center located in Chengdu. The demographic and clinical characteristics of our cohort are shown in Table 1. The median age was 43 years (interquartile range, 31.8–51.0), and 56.6% were male. Fever occurred in 42.9% of patients from the earliest onset of symptom and in 29.5% on admission. The second most common symptom was cough (31.4%). Digestive symptoms, including vomiting and diarrhea, were present in 7.4% of cases. In our cohort, 25.9% had at least one coexisting disease (hypertension, diabetes, or chronic obstructive pulmonary disease).

Disease severity was considered mild in 323 patients and severe in 43 patients. Patients with severe disease were older than those with mild disease by a median of 6 years. More than 50% of severe patients had systolic blood pressure greater than 110 mmHg. Moreover, fever, cough, and dyspnea were more common in patients with severe disease than those with mild disease (58.1% vs. 40.9, 69.8% vs. 26.3, and 27.9% vs. 3.4%, respectively). Comorbidities were more prevalent in severe patients than mild patients, including hypertension (32.6% vs. 7.4%), cardiovascular disease (16.3% vs. 0.6%), diabetes (14.0% vs. 4.6%), chronic liver disease (9.3% vs. 1.2%), and chronic kidney disease (7.0% vs. 0.3%). More than 37% of severe patients had visited Wuhan or other COVID-affected areas in the past 14 days. However, the history of contact with febrile or COVID-19 patients was similar between the two groups.

### Selection of independent predictive factors

**Fig 1:**
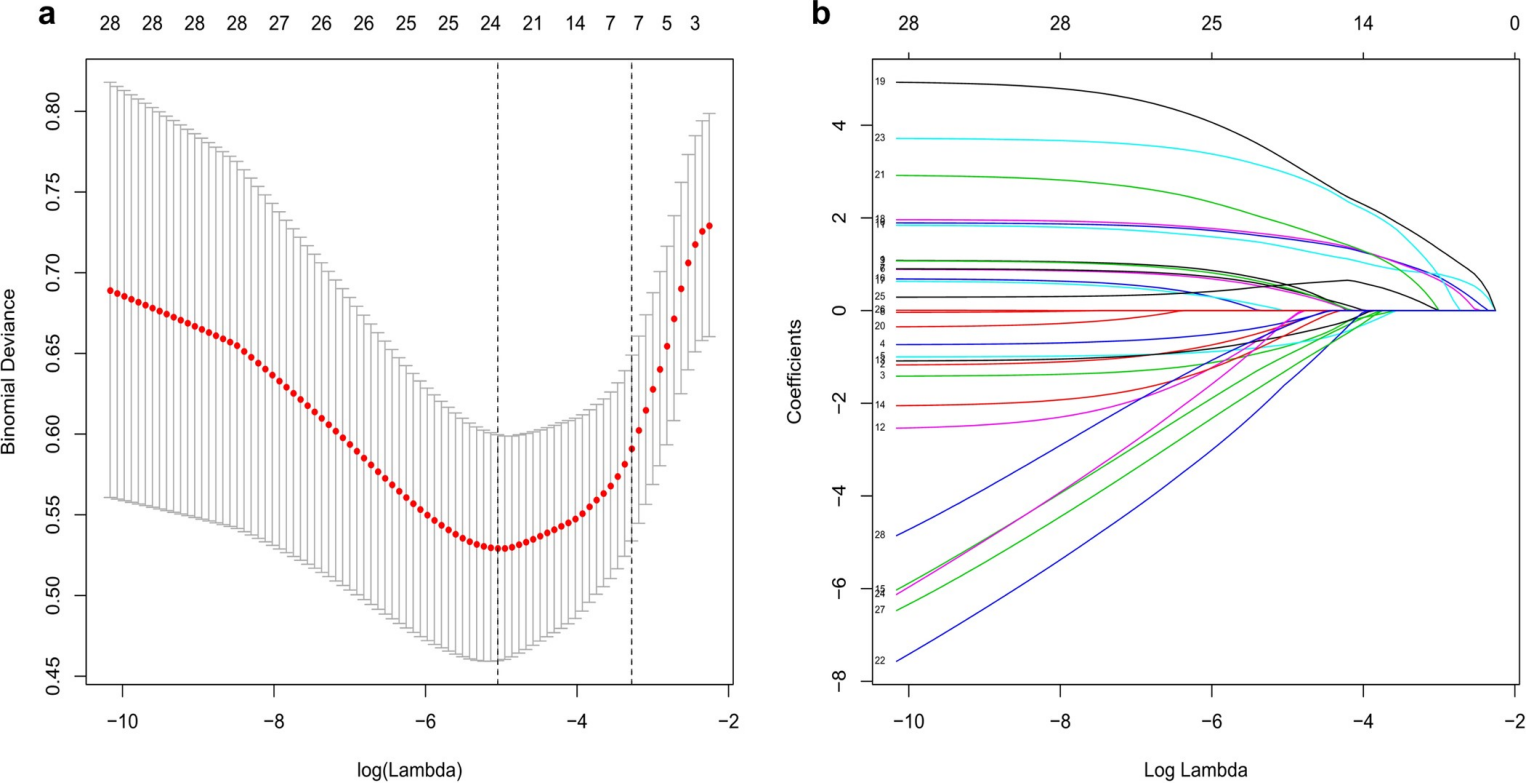
Selection of demographic and clinical features using the least absolute shrinkage and selection operator (LASSO) logistic regression model. (a). Selection of optimal parameters (lambda) from the LASSO model using five-fold cross-validation and minimum criteria. The partial likelihood deviance (binomial deviance) curve was plotted versus log(lambda). Dotted vertical lines were drawn at the optimal values using the minimum criteria and the 1 standard error of the minimum criteria (1-SE criteria). (b). LASSO coefficient profiles of 24 features. A coefficient profile plot was produced against the log(lambda) sequence. A vertical line was drawn at the value.

Based on demographics, clinical signs on admission, clinical symptoms, clinical risk factors, and exposure to infection, seven potential predictors with non-zero coefficients were selected in the LASSO logistic regression model (Fig 1A and 1B).

The selected predictors were body temperature on admission, cough, dyspnea, hypertension, cardiovascular disease, chronic liver disease, and chronic kidney disease. The results of the logistic regression analysis are shown in Table 2.

**Table 2:** Logistic regression analysis of the ability of each variable to predict the risk of severe COVID-19.

	Prediction model		
	*β*	Odds ratio (95% CI)	P-value
**Intercept**	**–4.524**	**0.011(0.000–0.085)**	**0.000**
NEWS score	1.029	2.800 (0.697–11.315)	0.145
Age	–1.188	0.305 (0.026–1.698)	0.243
**Temperature at admission**	**–2.776**	**0.062 (0.005–0.527)**	**0.017**
Respiratory rate	–0.762	0.467 (0.084–2.216)	0.355
Heart rate	–0.952	0.386 (0.079–1.414)	0.186
Oxygen saturation	0.837	2.310 (0.729–7.042)	0.143
Fever	0.891	2.437 (0.351–52.017)	0.448
Systolic blood pressure	0.867	2.380 (0.617–9.063)	0.203
**Cough**	**1.921**	**6.829 (2.288–22.185)**	**0.001**
**Dyspnea**	**2.027**	**7.594 (1.445–47.303)**	**0.020**
Chest pain	–2.688	0.068 (0.000–11.564)	0.562
Fatigue	–1.280	0.278 (0.029–1.721)	0.206
Muscle and joint pain	–2.087	0.124 (0.002–2.894)	0.254
Digestive symptoms	–16.402	0.000 (0.000–Inf)	0.993
Diabetes	0.549	1.732 (0.275–8.618)	0.524
**Hypertension**	**1.909**	**6.748 (2.026–23.200)**	**0.002**
**Cardiovascular disease**	**4.817**	**123.628 (7.985–7441.609)**	**0.004**
**Chronic liver disease**	**2.817**	**16.719 (1.217–258.169)**	**0.038**
Cerebrovascular disease	–19.079	0.000 (NA–Inf)	0.997
**Chronic kidney disease**	**3.676**	**39.492 (1.877–1107.008)**	**0.013**
Malignancy	–18.392	0.000 (NA–Inf)	0.999
Recently visited Wuhan Province or other COVID-affected areas	0.463	1.589 (0.460–5.429)	0.458
Contact with COVID-19 patients	–17.023	0.000 (NA–Inf)	0.993
Cluster of pneumonia	–15.673	0.000 (NA–Inf)	0.994

NEWS, national early warning score.

### Building and validating a prediction nomogram model

**Fig 2:**
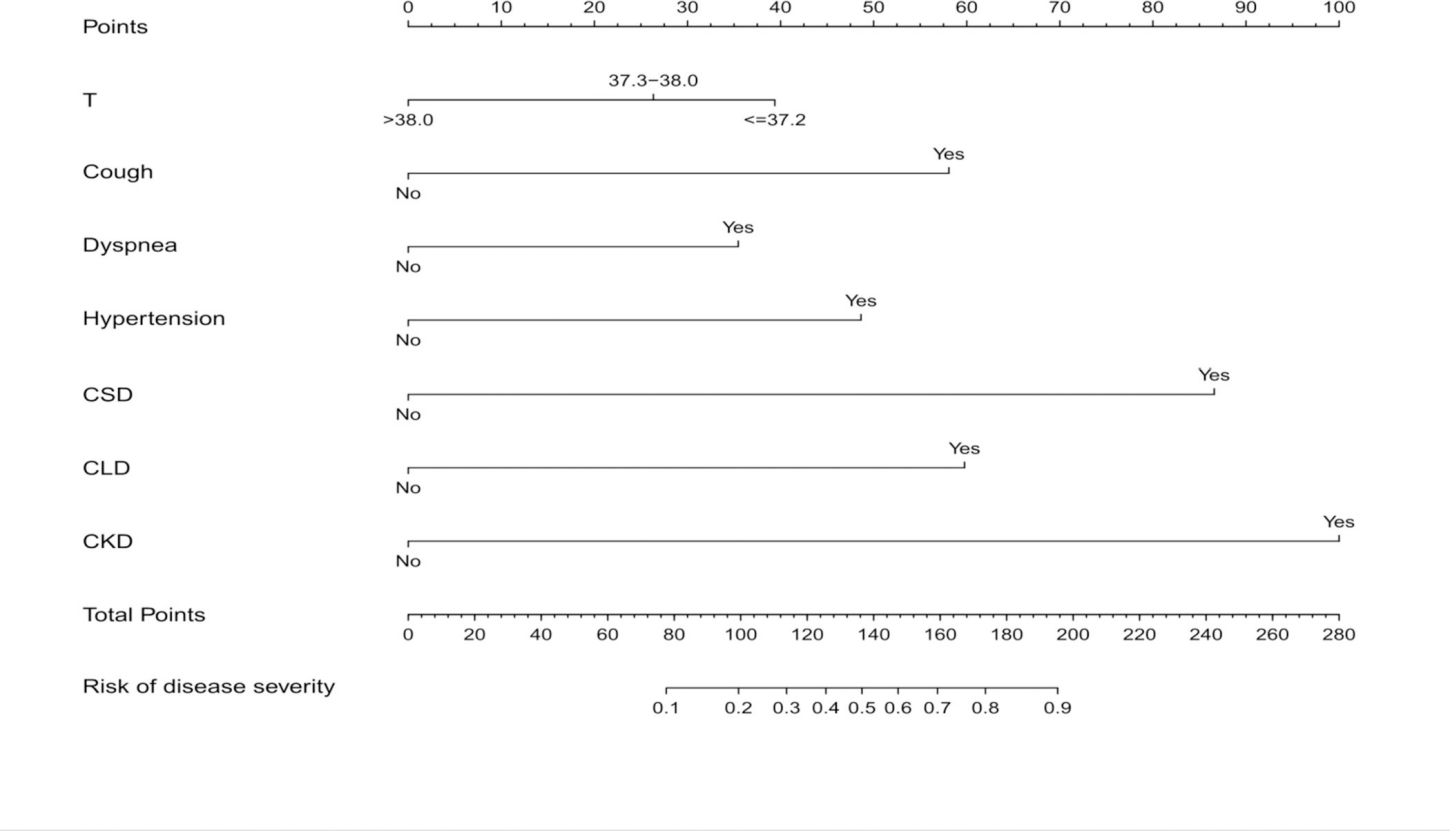
Development of a nomogram for predicting severe COVID-19. The nomogram included body temperature at admission, oxygen saturation, cough, dyspnea, hypertension, cardiovascular disease, chronic liver disease, and chronic kidney disease. The nomogram summed the scores for each scale and variable. The total score on each scale indicated the risk of severe COVID-19.

The nomogram used for predicting severe COVID-19 was formulated using significant independent factors, including body temperature at admission, cough, dyspnea, hypertension, cardiovascular disease, chronic liver disease, and chronic kidney disease. The nomogram showed that the best predictor of severity was comorbidity, including chronic kidney disease, cardiovascular disease, and chronic liver disease. Each variable was assigned a score according to the demographic and clinical characteristics of each patient, and the total score was computed by summing individual scores. Patient severity probabilities were also obtained from the nomogram (Fig 2).

**Fig 3:**
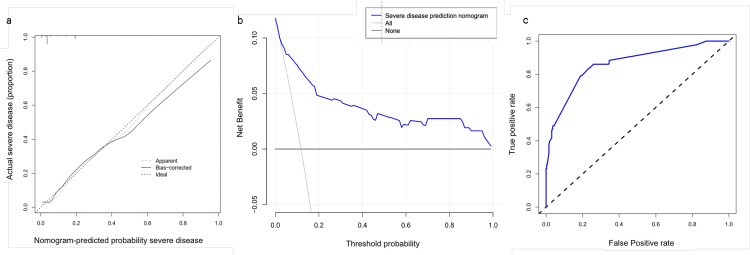
a. Calibration curves of the nomogram for predicting severe COVID-19. Data on predicted and actual disease severity were plotted on the x- and y-axis, respectively. The diagonal dotted line indicates the ideal nomogram, in which actual and predicted probabilities are identical. The solid line indicates the actual nomogram, and a better fit to the dotted line indicates a better calibration. b. Decision curves of the nomogram predicting severe COVID-19. The x-axis represents threshold probabilities and the y-axis measures the net benefit calculated by adding true positives and subtracting false positives. c. Receiver-operating characteristic curve of the nomogram for predicting severe COVID-19.

The C-index of the nomogram was 0.863 (95% CI, 0.801–0.925) in our model and 0.839 by bootstrapping analysis, suggesting that the model had good discriminative ability. The calibration plots of the nomogram showed that the agreement between predicted and observed severity was optimal (Fig 3A). In addition, DCA showed that the predictive model had significant net benefits for almost all threshold probabilities at different time points, demonstrating the potential clinical benefit of the predictive model (Fig 3B). The AUC of the nomogram was 0.862, indicating improved survival prediction compared with the nomogram model (Fig 3C).

## Discussion

We developed and validated a prediction nomogram based on clinical features to identify patients who might develop severe disease. The nomogram included vital signs, symptoms, and comorbidities, and showed good discrimination and calibration. Our model is useful to predict severe COVID-19. Traditional evaluation scoring tools, including NEWS, qSOFA, and CURB-65, are adopted to assess disease severity in emergency departments[23, 25, 26]. However, there is no evidence that these tools are useful for the early assessment of COVID-19 severity. Compared with other diseases, COVID-19 progresses faster, and severity cannot be identified promptly. The early symptoms of COVID-19 are more insidious, the disease progresses faster, and early detection is challenging. Therefore, our nomogram is a convenient and valuable clinical tool for predicting severe COVID-19.

Previous studies have shown that age is an important independent prognostic factor in patients with severe infection diseases, such as severe acute respiratory syndrome (SARS) and Middle East respiratory syndrome (MERS) [20, 26]. It has been demonstrated that prognosis in older COVID-19 patients, especially those aged >65 years, was worse than in younger patients [1, 5]. In our study, the severe group was older than the non-severe group. However, age was not an independent predictive factor for severe disease.

Underlying diseases are more prevalent in severe COVID-19 patients than in mild patients, and the most common comorbidities are hypertension, diabetes, and coronary heart disease[11, 27]. In the univariate analysis, hospital mortality was significantly higher in patients with underlying diseases (i.e., diabetes and coronary heart disease) than in patients without these comorbidities. To the best of our knowledge, no other studies have evaluated the relationship between underlying diseases and COVID-19 severity. Our study found that chronic cardiovascular disease, hypertension, kidney disease, and liver disease were risk factors for the development of severe illness.

COVID-19 impairs the function of multiple organs, including the heart, liver, and kidneys. Existing research suggests that angiotensin‑converting enzyme 2 (ACE2) may be a functional receptor for SARS-CoV-2 entry into human cells, and the virus may increase pulmonary vascular permeability and induce acute lung injury by down-regulating ACE2 expression and increasing angiotensin II levels[28–30]. ACE2 receptors are highly expressed in cells of the bronchial epithelium, alveoli (type 2 cells), myocardium, renal proximal tubule epithelium, bladder epithelium, esophagus, and ileum, suggesting that SARS-Cov-2 infection not only affects the respiratory system, but may also affect the circulatory, urinary, and digestive systems[31]. Severe patients have multiple organ damage, potentially leading to multiple organ failure. However, additional studies are needed to confirm that patients with underlying diseases (i.e., cardiovascular disease or kidney disease) infected with SARS-Cov-2 will accelerate this series of processes and their underlying mechanisms.

In the early stages of COVID-19, the diversity of symptoms and imaging manifestations limit diagnosis[32–34]. Fever and cough are common, and gastrointestinal symptoms are rare in COVID-19[35]. No fever is more common than SARS and MERS in patients with early stage COVID-19[11]. Therefore, patients without fever may be undiagnosed. This study found that body temperature higher than 37.3°C was not a risk factor in COVID-19, and patients without fever in the early stage of the disease had a higher risk of developing severe conditions. The reason is that fever may encourage the patient to seek medical treatment promptly, allowing early disease detection and implementation of medical interventions. In addition, fever can inhibit the reproduction or growth of the virus; however, this process is complex, and the effect of fever on this parameter should be better investigated.

The clinical data obtained on admission are included in the COVID-19 early warning system, which is simple, practical, reliable, and fast. This system was used to assess the risk of developing critical illness in the emergency department and allows medical staff to intervene at an early stage and determine their treatment location and the type of intervention. This system is more practical to evaluate COVID-19 patients than other scoring tools.

Our study has some limitations. First, the design was retrospective. Second, some cases had incomplete data on symptoms, laboratory tests, and imaging examinations, given the variation in the structure of electronic databases across different participating hospitals and an urgent data extraction schedule. Third, some patients were not discharged during the study period, and final prognosis could not be determined. Fourth, the model verification method used internal random verification. Fifth, although the AUC was high (0.863) and 95% CI was adequate (0.801–0.925), the number of severe cases was small; therefore, future studies with larger sample sizes are warranted to validate our results. Sixth, severe patients were older than non-severe patients, and this difference in age may be a confounding factor. Seventh, although the study is multicenter, the results cannot be generalized to other populations.

## Conclusion

We established an early warning model incorporating clinical characteristics that could be quickly obtained on hospital admission. This model can be conveniently used to facilitate the predict the individual risk of severe COVID-19 and help identify patients who might develop severe disease at early stage with convenience.

## References

[1] Zhu N, Zhang D, Wang W, Li X, Yang B, Song J, et al A Novel Coronavirus from Patients with Pneumonia in China, 2019. N Engl J Med. 2020;382(8):727–33. Epub 2020/01/25. 10.1056/NEJMoa2001017 . 31978945 PMC7092803

[2] Hui DS, E IA, Madani TA, Ntoumi F, Kock R, Dar O, et al The continuing 2019-nCoV epidemic threat of novel coronaviruses to global health—The latest 2019 novel coronavirus outbreak in Wuhan, China. Int J Infect Dis. 2020;91:264–6. Epub 2020/01/19. 10.1016/j.ijid.2020.01.009 . 31953166 PMC7128332

[3] Zhou P, Yang XL, Wang XG, Hu B, Zhang L, Zhang W, et al A pneumonia outbreak associated with a new coronavirus of probable bat origin. Nature. 2020;579(7798):270–3. Epub 2020/02/06. 10.1038/s41586-020-2012-7 32015507 PMC7095418

[4] Li Q, Guan X, Wu P, Wang X, Zhou L, Tong Y, et al Early Transmission Dynamics in Wuhan, China, of Novel Coronavirus-Infected Pneumonia. N Engl J Med. 2020 Epub 2020/01/30. 10.1056/NEJMoa2001316 . 31995857 PMC7121484

[5] Koonin LM. Novel coronavirus disease (COVID-19) outbreak: Now is the time to refresh pandemic plans. J Bus Contin Emer Plan. 2020;13(4):1–15. Epub 2020/03/13. . 32438951

[6] Huang C, Wang Y, Li X, Ren L, Zhao J, Hu Y, et al Clinical features of patients infected with 2019 novel coronavirus in Wuhan, China. Lancet. 2020;395(10223):497–506. Epub 2020/01/28. 10.1016/S0140-6736(20)30183-5 . 31986264 PMC7159299

[7] Guan WJ, Ni ZY, Hu Y, Liang WH, Ou CQ, He JX, et al Clinical Characteristics of Coronavirus Disease 2019 in China. N Engl J Med. 2020 Epub 2020/02/29. 10.1056/NEJMoa2002032 . 32109013 PMC7092819

[8] Novel Coronavirus Pneumonia Diagnosis and Treatment Plan (Provisional 6th Edition) [cited 2020 April 17th]. Available from: http://www.gov.cn/zhengce/zhengceku/2020-03/04/5486705/files/ae61004f930d47598711a0d4cbf874a9.pdf.

[9] Chen N, Zhou M, Dong X, Qu J, Gong F, Han Y, et al Epidemiological and clinical characteristics of 99 cases of 2019 novel coronavirus pneumonia in Wuhan, China: a descriptive study. Lancet. 2020;395(10223):507–13. Epub 2020/02/03. 10.1016/S0140-6736(20)30211-7 . 32007143 PMC7135076

[10] Chan JF, Yuan S, Kok KH, To KK, Chu H, Yang J, et al A familial cluster of pneumonia associated with the 2019 novel coronavirus indicating person-to-person transmission: a study of a family cluster. Lancet. 2020;395(10223):514–23. Epub 2020/01/28. 10.1016/S0140-6736(20)30154-9 . 31986261 PMC7159286

[11] Wang D, Hu B, Hu C, Zhu F, Liu X, Zhang J, et al Clinical Characteristics of 138 Hospitalized Patients With 2019 Novel Coronavirus-Infected Pneumonia in Wuhan, China. JAMA. 2020 Epub 2020/02/08. 10.1001/jama.2020.1585 32031570 PMC7042881

[12] Yang W, Cao Q, Qin L, Wang X, Cheng Z, Pan A, et al Clinical characteristics and imaging manifestations of the 2019 novel coronavirus disease (COVID-19):A multi-center study in Wenzhou city, Zhejiang, China. J Infect. 2020 Epub 2020/03/01. 10.1016/j.jinf.2020.02.016 . 32112884 PMC7102539

[13] Iasonos A, Schrag D, Raj GV, Panageas KS. How to build and interpret a nomogram for cancer prognosis. J Clin Oncol. 2008;26(8):1364–70. 10.1200/JCO.2007.12.9791 PubMed PMID: WOS:000254178300028. 18323559

[14] Wu JL, Qiu JT, Jiang WX, Qiu JW, Zhang L, Zhao R, et al Development and validation of a nomogram predicting the probability of type a aortic dissection at a diameter below 55 mm: A retrospective cohort study. Int J Surg. 2018;60:266–72. 10.1016/j.ijsu.2018.11.024 PubMed PMID: WOS:000453422900035. 30496867

[15] Chang, Lin M, Wei L, Xie L, Zhu G, Dela Cruz CS, et al Epidemiologic and Clinical Characteristics of Novel Coronavirus Infections Involving 13 Patients Outside Wuhan, China. JAMA. 2020 Epub 2020/02/08. 10.1001/jama.2020.1623 32031568 PMC7042871

[16] Corona- virus disease (COVID-19) technical guid- ance: laboratory testing for 2019-nCoV in humans [Internet]. 2019. Available from: (https://www.who.int/emergencies/diseases/novel-coronavirus-2019/technical-guidance/laboratory-guidance).

[17] Clinical management of severe acute respiratory infection when novel coronavirus (2019-nCoV) infection is suspected: interim guidance. [Internet]. 2020 Available from: (https://www.who.int/docs/default-source/coronaviruse/clinical-management-of-novel-cov.pdf).

[18] Teasdale GM. National early warning score (NEWS) is not suitable for all patients. BMJ. 2012;345:e5875 Epub 2012/09/07. 10.1136/bmj.e5875 . 22951554

[19] Metlay JP, Waterer GW, Long AC, Anzueto A, Brozek J, Crothers K, et al Diagnosis and Treatment of Adults with Community-acquired Pneumonia An Official Clinical Practice Guideline of the American Thoracic Society and Infectious Diseases Society of America. Am J Resp Crit Care. 2019;200(7):E45–E67. 10.1164/rccm.201908-1581ST PubMed PMID: WOS:000488777100003. 31573350 PMC6812437

[20] Sauerbrei W, Royston P, Binder H. Selection of important variables and determination of functional form for continuous predictors in multivariable model building. Stat Med. 2007;26(30):5512–28. 10.1002/sim.3148 PubMed PMID: WOS:000252595600014. 18058845

[21] Friedman J, Hastie T, Tibshirani R. Regularization Paths for Generalized Linear Models via Coordinate Descent. J Stat Softw. 2010;33(1):1–22. 10.18637/jss.v033.i01 PubMed PMID: WOS:000275203200001. 20808728 PMC2929880

[22] Kidd AC, McGettrick M, Tsim S, Halligan DL, Bylesjo M, Blyth KG. Survival prediction in mesothelioma using a scalable Lasso regression model: instructions for use and initial performance using clinical predictors. Bmj Open Respir Res. 2018;5(1). doi: UNSP e000240 10.1136/bmjresp-2017-000240 PubMed PMID: WOS:000438737600003. 29468073 PMC5812388

[23] Niederman MS, Mandell LA, Anzueto A, Bass JB, Broughton WA, Campbell GD, et al Guidelines for the management of adults with community-acquired pneumonia. Diagnosis, assessment of severity, antimicrobial therapy, and prevention. Am J Respir Crit Care Med. 2001;163(7):1730–54. Epub 2001/06/13. 10.1164/ajrccm.163.7.at1010 . 11401897

[24] Alba AC, Agoritsas T, Walsh M, Hanna S, Iorio A, Devereaux PJ, et al Discrimination and Calibration of Clinical Prediction Models Users' Guides to the Medical Literature. Jama-J Am Med Assoc. 2017;318(14):1377–84. 10.1001/jama.2017.12126 PubMed PMID: WOS:000412838900021. 29049590

[25] Ito A, Ishida T, Tokumasu H, Washio Y, Yamazaki A, Ito Y, et al Prognostic factors in hospitalized community-acquired pneumonia: a retrospective study of a prospective observational cohort. BMC Pulm Med. 2017;17(1):78 Epub 2017/05/04. 10.1186/s12890-017-0424-4 28464807 PMC5414343

[26] Niederman MS, Bass JB Jr., Campbell GD, Fein AM, Grossman RF, Mandell LA, et al Guidelines for the initial management of adults with community-acquired pneumonia: diagnosis, assessment of severity, and initial antimicrobial therapy. American Thoracic Society. Medical Section of the American Lung Association. Am Rev Respir Dis. 1993;148(5):1418–26. Epub 1993/11/01. 10.1164/ajrccm/148.5.1418 . 8239186

[27] Guo L, Wei D, Zhang X, Wu Y, Li Q, Zhou M, et al Clinical Features Predicting Mortality Risk in Patients With Viral Pneumonia: The MuLBSTA Score. Front Microbiol. 2019;10:2752 Epub 2019/12/19. 10.3389/fmicb.2019.02752 31849894 PMC6901688

[28] Xu Z, Shi L, Wang Y, Zhang J, Huang L, Zhang C, et al Pathological findings of COVID-19 associated with acute respiratory distress syndrome. Lancet Respir Med. 2020 Epub 2020/02/23. 10.1016/S2213-2600(20)30076-X . 32085846 PMC7164771

[29] Kuba K, Imai Y, Rao S, Gao H, Guo F, Guan B, et al A crucial role of angiotensin converting enzyme 2 (ACE2) in SARS coronavirus-induced lung injury. Nat Med. 2005;11(8):875–9. Epub 2005/07/12. 10.1038/nm1267 . 16007097 PMC7095783

[30] Imai Y, Kuba K, Rao S, Huan Y, Guo F, Guan B, et al Angiotensin-converting enzyme 2 protects from severe acute lung failure. Nature. 2005;436(7047):112–6. Epub 2005/07/08. 10.1038/nature03712 . 16001071 PMC7094998

[31] Oudit GY, Crackower MA, Sarao R, Yagil Y, Chappell M, Backx PH, et al Angiotensin-converting enzyme 2 is an essential regulator of heart function. Circulation. 2002;106(19):315-. PubMed PMID: WOS:000179142701618. 10.1038/nature00786 12075344

[32] Xu X, Yu CC, Zhang LG, Luo LP, Liu JX. Imaging features of 2019 novel coronavirus pneumonia. Eur J Nucl Med Mol I. 2020 10.1007/s00259-020-04720-2 PubMed PMID: WOS:000516170700001. 32060619 PMC7079932

[33] Munster VJ, Koopmans M, van Doremalen N, van Riel D, de Wit E. A Novel Coronavirus Emerging in China—Key Questions for Impact Assessment. New Engl J Med. 2020;382(8):692–4. 10.1056/NEJMp2000929 PubMed PMID: WOS:000517119800003. 31978293

[34] Yang X, Yu Y, Xu J, Shu H, Xia J, Liu H, et al Clinical course and outcomes of critically ill patients with SARS-CoV-2 pneumonia in Wuhan, China: a single-centered, retrospective, observational study. Lancet Respir Med. 2020 Epub 2020/02/28. 10.1016/S2213-2600(20)30079-5 . 32105632 PMC7102538

[35] Sun P, Qie S, Liu Z, Ren J, Li K, Xi J. Clinical characteristics of 50 466 hospitalized patients with 2019-nCoV infection. J Med Virol. 2020. Epub 2020/02/29. 10.1002/jmv.25735 . 32108351 PMC7228255

